# Laser induced diffuse reflectance imaging – Monte Carlo simulation of backscattering measured on the surface

**DOI:** 10.1016/j.mex.2020.100958

**Published:** 2020-06-18

**Authors:** László Baranyai

**Affiliations:** Leibniz Institute for Agricultural Engineering and Bioeconomy (ATB), Germany

**Keywords:** Photon trajectory, Light interaction, Spatial multispectral imaging

## Abstract

The Monte Carlo simulation algorithm of photon trajectory computation is implemented in object oriented R code. Diffuse reflectance, also called backscattering, is modeled in semi-infinite homogeneous media. Spatial photon flux leaving the surface of the media is collected. The profile of intensity along radii relative to the incident point is used to simulate measurement of computer vision systems. Four optical parameters of the media are used: absorption coefficient, scattering coefficient, anisotropy factor and refractive index. Five parameters are used to describe configuration of the vision system: number of photons, radius of circular light beam, limiting energy level of photons, radius of observed area, spatial resolution of the vision system.

• The incident angle of the light beam is included in the photon launch procedure. Initial direction is typically assumed to be normal with x,y,z coordinates of 0,0,1. In the proposed modification, initial move vector is calculated based on the incident angle and refractive index of the media. Additionally, elliptic distortion of the circular light beam on the surface is calculated based on the incident angle.
• Photon flux leaving media through the surface is corrected with Lambertian method to measure intensity captured by an imaging device in normal position.
• The software implementing the method is written in R language, the R code is available as standard package.

## Graphical abstract

**Figure:**
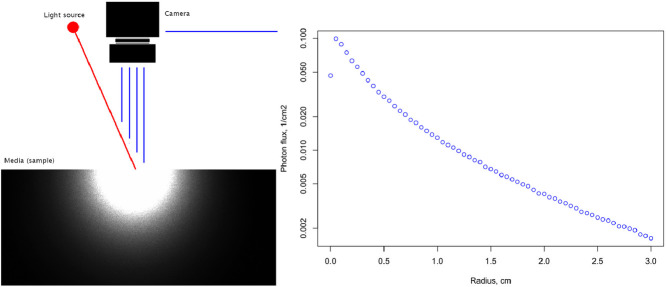

Specifications Table

**Table:** 

Subject Area	*Agricultural and Biological Sciences Computer Science*
More specific subject area:	*Optical properties of biological tissue*
Method name:	*Monte Carlo simulation of diffuse reflectance measured on the surface*
Name and reference of original method	*The code presented in this paper implements stochastic Monte Carlo algorithm to simulate light distribution in media and its measurement on the surface. The computational methods are inspired by the literature and primarily:* • Francesc Salvat: PENELOPE-2014 • A Code System for Monte Carlo Simulation of Electron and Photon Transport. Workshop Barcelona, Spain 29 June-3 July 2015 (NEA/NSC/DOC(2015)3) • Jacques, S.L., 1998. Light distributions from point, line and plane sources for photo-chemical reactions and fluorescence in turbid biological tissues. Photochemistry and Photobiology 67 (1), 23–32.
Resource availability	*Object oriented R code (*www.r-project.org*) was written and an R package has been created.*

## Method details

The name Monte Carlo (MC) indicates stochastic behavior of computation. The simulation of diffuse reflectance, also known as backscattering, follows photon pathways inside medium and summarizes photon flux leaving the surface. The simulation assumes semi-infinite homogeneous media and an imaging device above the surface to collect photons and measure spatial intensity distribution. Detailed description of the photon trajectory computation algorithm and software libraries are available in Fortran language (with the name of PENELOPE) [1,2] and ANSI C language (with the name of MCML and CONV) [3,4]. This implementation uses variable names similar to the ANSI C code.

**Fig. 1:**
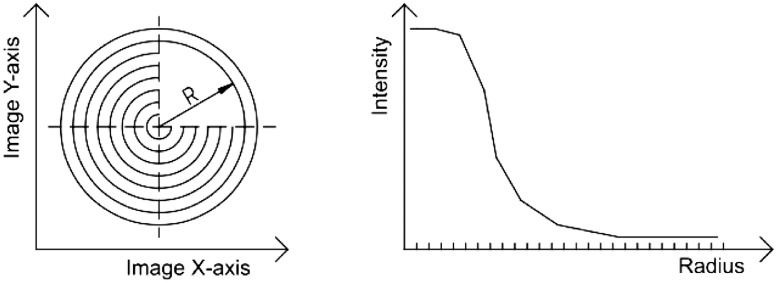
Radial averaging pattern of concentric rings (left) and typical shape of acquired intensity profile (right). R is the radius measured from incident point.

Computer vision systems measure spatial intensity distribution relative to the incident point of the light beam [5,6]. The light beam injects photons into the media and surrounding area got illuminated by diffuse reflectance. Intensity is typically measured in concentric rings of 1 pixel width (Fig. 1). The captured intensity profile is very similar to the result of Monte Carlo simulation. Monte Carlo simulation can be used in inverse modeling. Estimation functions, established based on the observed results of simulation, can make predictions in vision systems [7,8].

The measured photon flux and the shape of the intensity profile (Fig. 1) depends on the optical properties of the media. These parameters are μ_a_ absorption coefficient (cm^−1^), μ_s_ scattering coefficient (cm^−1^), *g* anisotropy factor and *n* refractive index of media. The reduced scattering coefficient (Eq. (1)) is reported in many publications as simplified parameter of the theoretical model [9].

(1) $$\mu_{s}\text{’}=\left(1-g\right)\mu_{s}$$

**Fig. 2:**
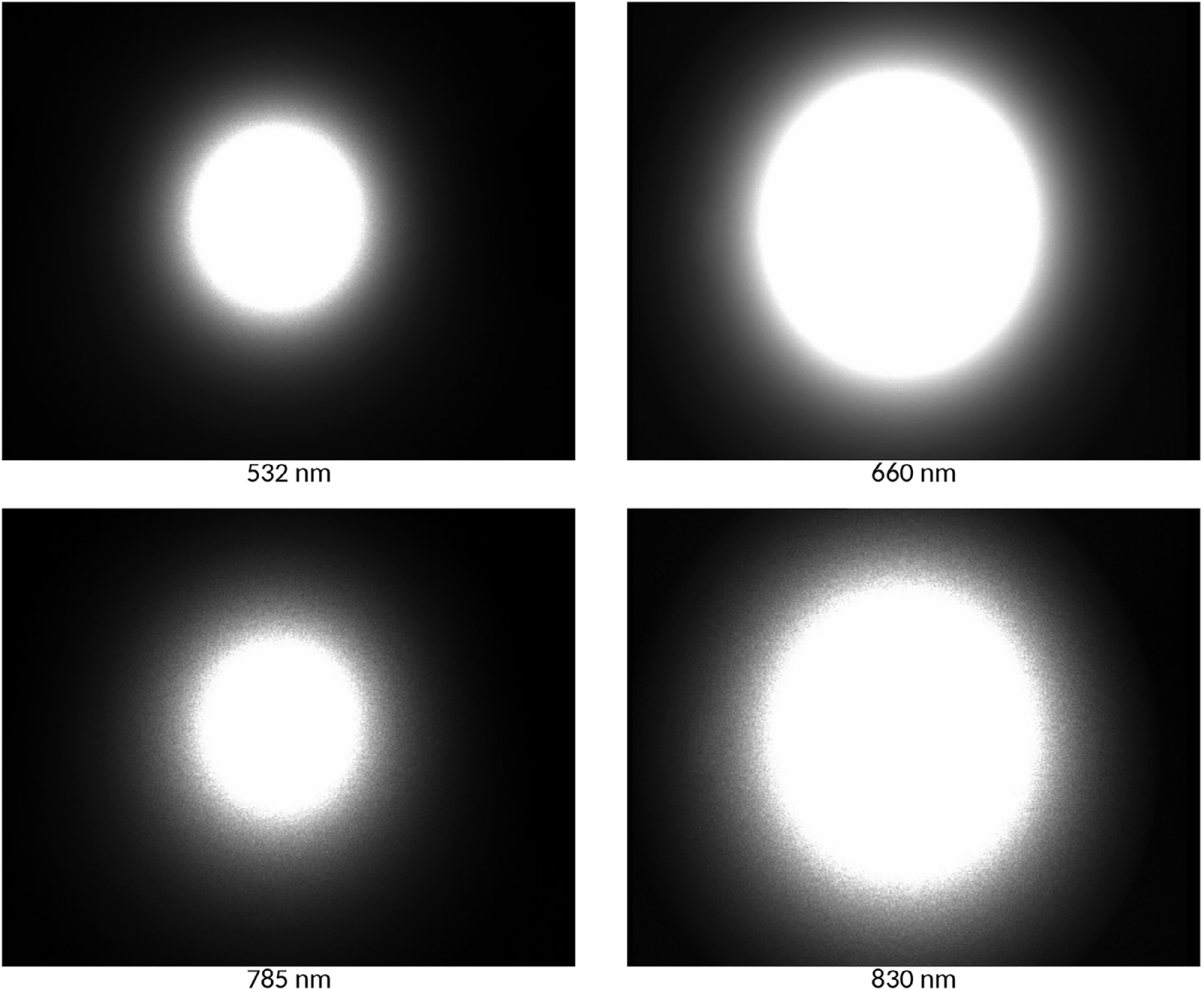
Laser induced diffuse reflectance of the same object at different wavelengths. Reference material of μ*_a_* = 0.1056 cm^−1^ and μ_s_‘ = 2.529 cm^−1^ at 680 nm.

This simplification makes validation more difficult, since many different combinations of *g* and μ_s_ can result the same μ_s_’ reduced scattering coefficient. Media could be assumed isotropic (*g* = 0) but biological materials are reported to scatter forward *g* > 0.6 [10]. Additionally, absorption and scattering coefficients may differ by wavelength. The effect of wavelength on optical measurement is demonstrated in Fig. 2, where the same object can be observed with diffuse reflectance induced at multiple wavelengths.

The computational parameters of the simulation include number of photons, radius of circular light beam, limiting energy level of photons, radius of observed area, spatial resolution of the vision system. The number of photons significantly affects computation time, therefore the minimum sufficient number shall be adjusted. The noise of the calculated intensity profile decreases with increasing number of photons. Based on the power of the light source and wavelength of emitted light, required number of photons can be calculated using Planck's law (Eq. (2)):

(2) $$E=h\nu =h\frac{C_{0}}{n\lambda }$$

where *E* is the photon energy, *h* is the Planck's constant, *v* is the frequency, *C_0_* is the velocity of light in vacuum, λ is the wavelength of light and *n* is the refractive index of media. For example, 1 s light pulse of 670 nm wavelength of 3 mW power in media of *n* = 1.4 result in 1.42 × 10^16^ photons. This number can decrease if integration time of the imaging device of computer vision system was considered. Based on time resolved calculations, the 1 ns pulse length was found to be sufficient for simulation of light penetration into apple [5].

The hardware parameters of the computer vision system are constant for the same setup, such as image resolution, beam radius. The parameter limiting energy shall be low enough to allow drop low energy photons without significant computation error. The simulation can be initialized with optical parameters of media and parameters of the computer vision system. The R code to load package and initialize simulation object is presented in Table 1. Absorption and scattering coefficients are expected in cm^−1^ unit.

**Table 1:** Loading library and configuration of simulation object MCBS in R language.

## Load library
library("MCBackscattering")
## Apple tissue properties according to
## Qin and Lu (2006) DOI: 10.13031/2013.20862
cfgMedia <- c(
0.63, # absorption 1/cm, 670 nm
30, # scattering 1/cm, 670 nm
0, # isotropic tissue assumed
1.4) # refractive index
## Computer vision system and simulation parameters
cfgSimulation <- c(
1e7, # 10 million photons
0.05, # 1 mm diameter (0.05 cm radius) laser light beam
1e-9, # limiting energy level
3, # 3 cm radius is computed
0.01) # 0.01 cm/pixel resolution
apple <- MCBS(cfgMedia,cfgSimulation)

Running simulation with this implementation does not require additional preparation. One function is made to perform all computations (Table 2) and result can be presented on chart or extracted as table.

**Table 2:** Usage of simulation function in R language. Code also shows profile on chart and saves data into file.

## Run simulation with default incident angle
apple <- Simulation(apple)
## Show intensity profile
Chart(apple)
## Save results into file with data table
write.table(Export(apple),"apple.dat")

**Fig. 3:**
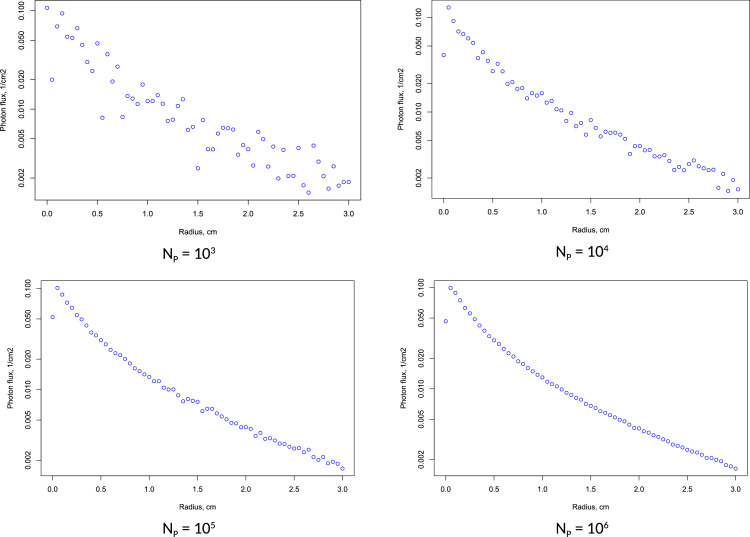
Effect of the number of photons (N_P_) on simulation result (μ*_a_* = 0.1056 cm^−1^, μ*_s_* = 2.529 cm^−1^, *g* = 0, *n* = 1.35).

Low number of photons in simulation obtain significant noise on the intensity profile. Low intensity values far from incident point may have higher noise due to the lower number of photons in that area. According to our experiences, the minimum recommended number of photons is 10^6^. The effect of the number of photons is presented on Fig. 3. Simulation of absorbed energy inside media can require less photons compared to diffuse reflection on surface, because the number of trajectories leaving the media on observed area is lower.

The simulation function (Table 2) has an optional second parameter, the incident angle of light beam. This value is used when photon is launched into media. If parameter is missing, default value of 0° is assumed. The normal position, above incident point, is occupied by the camera or other imaging device. Therefore, light source cannot be placed in the same direction. Measurement geometry of low incident angle is recommended to utilize large amount of photons. On the other hand, incident angle should be large enough to avoid direct reflection into the camera. The incident angle of 10–20° is recommended. The incident angle can be used with simulation function (Table 3).

**Table 3:** Usage of simulation function in R language with 15° incident angle. Code also saves data into table.

## Run simulation with 15 deg incident angle
apple <- Simulation(apple,15)
# Save results into file with data table
write.table(Export(apple),"apple.dat")

Computational methods presented in literature use normal direction, perpendicular to surface[1], [2], [3], [4],9]. Due to the limitations of hardware setup, this should be reconsidered.

Setup function (Table 4) was made to initialize computation at the beginning of the simulation. Container is created for results and derived parameters, such as albedo and critical angle, are calculated. The computation of simulation function may take long since it follows all photon trajectories. In order to accelerate procedure, maximum trajectory length and random launch position of all photons are adjusted, as well.

**Table 4:** Setup function implementation in R language to prepare containers, calculate parameters.

## Initialize computation
Setup <- function(myObject) {UseMethod("Setup",myObject)}
Setup.MCBS <- function(myObject)
{
# container for intesity profile
myObject$heat <- rep(0,1 + round(myObject$radius / myObject$rpx))
# transport albedo = intensity decrease after interaction event
myObject$albedo <- myObject$mu_s / (myObject$mu_*s*+ myObject$mu_a)
# specular reflection
myObject$rs <- ((myObject$n - 1) / (myObject$n + 1))^2
# critical angle
myObject$cangle <- sqrt(1.0 - 1.0/(myObject$n^2))
# maximum trajectory length in events
myObject$MAXLEN <- round(log(myObject$limit)/log(myObject$albedo))+1
# beam start position, radius and angle
myObject$rbr <- myObject$beamr*sqrt(runif(myObject$photons))
myObject$rba <- runif(myObject$photons,min=0,max=2*pi)
# reset summary values
myObject$rd <- 0
myObject$bit <- 0

return(myObject)
}

A randomize function (Table 5) was made with the purpose to accelerate computation and generate random numbers in advance for each photon. Computation later can refer to the prepared vectors of random numbers instead of calling the random number generation function each time.

**Table 5:** Randomize function implementation in R language to generate random numbers for single photon.

## Fill random data for single photon trajectory
Randomize <- function(myObject) {UseMethod("Randomize",myObject)}
Randomize.MCBS <- function(myObject)
{
# maximum trajectory length is computed to MAXLEN
# random numbers are selected from uniform distribution
# move length, 0–1
myObject$rmv <- runif(myObject$MAXLEN)
# absorption roulette, 0–1
myObject$rabs <- runif(myObject$MAXLEN)
# new direction after scattering, from −1 to +1
myObject$rx1 <- runif(myObject$MAXLEN,min=−1,max=1)
myObject$rx2 <- runif(myObject$MAXLEN,min=−1,max=1)
# Heyney-Greenstein phase function random variable, 0–1
myObject$rmu <- runif(myObject$MAXLEN)

return(myObject)
}

Photon trajectory computation has five steps:

• launch photon
• move in media
• bounce if photon leaves media through surface
• absorb energy
• scatter in media

Common computational method is modified in launch and bounce. Launch is considering incident angle and its effects, bounce collects photon weights on surface. All steps are available as separate functions to allow users build customized procedure.

The launch function has an optional second parameter, the incident angle. Initial direction inside media is calculated on the basis of the incident angle and refractive index (Eq. (3)).

(3) $$\phi_{2}=sin^{-1}\left(\frac{sin\phi_{1}}{n_{2}}\right)$$

where ϕ_1_ is the incident angle in air, ϕ_2_ is the refracted angle in media and *n_2_* is the refractive index of the media. When photon is emitted from light source, its weight is 1. The photon weight decreases first time during interaction with surface. Photons entering media are usually considered to have normal start direction (u,v,w) = (0,0,1). This direction is changed according to the incident angle. In media of *n* = 1.4, start angle is in the range of 7.12° – 14.14° for recommended incident angle range of 10° – 20° Additionally, due to the rotation of the incident light beam, its circular cross section is elliptic on the surface. This elliptical distortion is calculated on the launch position. The start position of the photon on the surface (x,y,z = 0) is calculated randomly within the area of the circular light beam, using uniform distribution [11]. Elliptic distortion affects this position with the y coordinate. The R language implementation of launch function is presented in Table 6.

**Table 6:** Launch function implementation in R language to start photon trajectory from boundary.

## Launch single photon within beam
# Parameter:
# iAngle = incident angle, default = 0 deg (relative to normal)
Launch <- function(myObject,iAngle=0) {UseMethod("Launch",myObject)}
Launch.MCBS <- function(myObject,iAngle=0)
{
# initial photon weight
myObject$weight <- 1.0 - myObject$rs
# internal angle after refraction
myAngle <- asin(sin(iAngle*pi/180)/myObject$n)
# incident direction
myObject$u <- 0
myObject$v <- sin(myAngle)
myObject$w <- cos(myAngle)
# start position
myObject$x <- myObject$rbr[myObject$idx] * cos(myObject$rba[myObject$idx])
myObject$y <- myObject$rbr[myObject$idx] * sin(myObject$rba[myObject$idx]) / cos(myAngle)
myObject$*z*<- 0

return(myObject)
}

During simulation, photons are identified with index number idx and trajectory moves for each photon are identified with index number midx. These indices are used to access containers of random numbers, such as launch position polar coordinates rbr for radius and rba for angle (Table 4,6). Bulk generation of random numbers is done for optimization of computation.

The second modified function compared to common algorithm is the bounce method. Photons leaving the surface are collected in this step. Photon position (x,y,z) is out of media if *z* < 0. When this occurs, moving direction (u,v,w) also points out of the media with *w* < 0. Moving direction is compared to critical angle to decide whether internal reflection happens or photon can leave media. Fresnel reflection is calculated to correct photon weight. Additionally, Lambertian correction is performed modeling the camera or imaging device in normal position above the surface. Photon flux is collected in vector heat, with spatial resolution. According to the R language, the first element of the vector has index 1, which belongs to the incident point of radius *r* = 0. Each element of the vector represent photon flux leaving the surface in the area of concentric ring around the incident point. The source code of the bounce function implemented in R language is presented in Table 7.

**Table 7:** Bounce function implementation in R language to collect surface photon flux.

## Bounce interaction with surface
Bounce <- function(myObject) {UseMethod("Bounce",myObject)}
Bounce.MCBS <- function(myObject)
{
myObject$w <- −1*myObject$w
myObject$*z*<- −1*myObject$z
# check for internal reflection, then nothing to do
if (myObject$w > myObject$cangle) {
t <- sqrt(1.0-(1.0-myObject$w^2)*myObject$n^2)
temp1 <- (myObject$w - myObject$n*t)/(myObject$w + myObject$n*t)
temp <- (t - myObject$n*myObject$w)/(t + myObject$n*myObject$w)
# Fresnel reflection
rf <- (temp1*temp1+temp*temp)/2.0
myObject$rd <- myObject$rd + (1.0-rf) * myObject$weight
# collect leaving photons by radius
# Lambertian correction to normal direction
lcc <- abs(myObject$w) / sqrt(myObject$u^2 + myObject$v^2 + myObject$w^2)
lcc <- myObject$n * sqrt(1.0-lcc^2)
if (lcc^2 > 1) {
# failsafe check
lcc <- 0
} else {
lcc <- sqrt(1.0 - lcc^2)
}
# compute radius
r <- sqrt(myObject$x^2 + myObject$y^2)
r <- round(r / myObject$rpx) + 1
if (r <= length(myObject$heat)) {
myObject$heat[r] <- myObject$heat[r] + lcc * (1.0-rf) * myObject$weight
}
# continue travel inside
myObject$weight <- myObject$weight - (1.0-rf) * myObject$weight;
}

return(myObject)
}

In order to receive the normalized photon flux in cm^−2^ unit, vector elements are divided by the corresponding surface area during post processing. The area of the concentric rings can be calculated using two equivalent equations (Eq. (4),(5).

(4) $$A=\pi \left({\left(r+dr\right)}^{2}-r^{2}\right)=\pi \left(2rdr+dr^{2}\right)$$

(5) $$A=2\pi \left(r+\frac{dr}{2}\right)dr$$

Where *A* is the area of the ring of *dr* width and *r* is the inner radius of the ring. The form of Eq. (5) is commonly used, but equations are equivalent and also calculated with similar speed. The R implementation is using formula Eq. (4).

Computations are accelerated to decrease simulation runtime. Trigonometric functions are substituted where it is possible using the following equation (Eq. (6)).

(6) $$\text{co}s^{2}\phi +\text{si}n^{2}\phi =1\;\text{and}\;\text{sin}\phi =\sqrt{1-\text{co}s^{2}\phi };\text{cos}\phi =\sqrt{1-\text{si}n^{2}\phi }$$

The simulation uses a lot of random numbers to launch photons and calculate their trajectory. Random numbers are generated in bulk for optimization. Start polar coordinates are generated in a vector with the length of the number of photons (Table 4). Trajectory random variables are generated in vectors with the length calculated from limiting energy and transport albedo (Table 5). Table 8 presents the random vectors used int the R implementation.

**Table 8:** Random vectors of R implementation of Monte Carlo simulation.

Vector name	Value range	Created by function	Used by function	Comment
rbr	0 – r_b_ *	Setup	Launch	Launch position polar coordinate, radius in light beam
rba	0 – 2π	Setup	Launch	Launch position polar coordinate, angle
rmv	0 – 1	Randomize	Move	Length of straight segment of trajectory
rabs	0 – 1	Randomize	Absorb	Photon survival decision is made after interaction
rx1, rx2	−1 – +1	Randomize	Scatter	New direction coordinates after scattering
rmu	0 – 1	Randomize	Scatter	Anisotropic scattering by Henyey-Greenstein function

* r_b_ is the radius of light beam.

The implemented code can be used according to presented sample (Table 2). Only two functions are required to perform simulation, the MCBS object constructor and Simulation to perform computation. Photon flux can be retrieved as intensity profile by Export function (Table 3). If one would like to make customized procedure, functions are available and Simulation function can be used as template.

Due to the interpreted R codes, computation consumes more time than compiled C software. Parallel processing may utilize multiple cores of processors on POSIX computer systems. Single core process simulation of 10^6^ photons took approximately 2 h on Intel i3 processor (3.83 GFLOPS/core).

## Method validation

Reference material of known optical properties was produced by PDW Analytics GmbH (Potsdam, Germany). The absorption coefficient and reduced scattering coefficient of solid phantom are known as μ*_a_* = 0.1056 cm^−1^ and μ_s_‘ = 2.529 cm^−1^ at 680 nm. The Monte Carlo simulation was repeated twice and used different combinations of anisotropy factor and scattering coefficient (resulting the same reduced scattering coefficient). Simulation parameters are listed in Table 9. The diffusion theory model [9] was also computed as reference. As a result of changing optical properties, rotation of simulated profiles can be observed [5,6]. Comparing the two simulations, results of isotropic media (*g* = 0) fit better to diffusion theory model in terms of correlation and RMSE (root mean squared error).

**Table 9:** Monte Carlo simulation parameters and results of validation.

Parameter	Isotropic media (*g* = 0)	Anisotropic media (*g* = 0.9)
Absorption coefficient (μ_a_, cm^−1^)	0.1056	0.1056
Scattering coefficient (μ_s_, cm^−1^)	2.529	25.29
Anisotropy factor (g)	0	0.9
Refractive index	1.35	1.35
Number of photons	10^6^	10^6^
Beam radius, cm	0.05	0.05
Limiting energy level for photons	10^−9^	10^−9^
Observed radius, cm	3	3
Spatial resolution, cm/pixel	0.05	0.05
Correlation with diffusion model	0.9903	0.9529
RMSE with diffusion model, cm^−2^	0.0534	0.1744

Backscattering images were recorded for the reference material at 660 nm wavelength (Fig. 2), using a laser module of 3 mW and a CCD camera (CV-A50IR, JAI Ltd., Japan) with zoom lens of 18–108 mm and f/2.5 (12VG1040 ASIR-SQ, Tamron Co. Ltd, Japan). The resolution of the images were 0.01205 cm/pixel. Images were recorded with 8 bit/pixel color depth, therefore pixel intensities ranged 0–255. All intensity profiles, measured and computed, were normalized to the range of 0–1 for comparison. The over saturated part near the incident point of the intensity profile was omitted from analysis. Comparison result of diffusion theory model as well as Monte Carlo simulation with measured signal is presented in Table 10.

**Table 10:** Comparison of computer vision system measurement with simulation results.

Parameter	Diffusion theory model	Monte Carlo simulation	
		Isotropic media (*g* = 0)	Anisotropic media (*g* = 0.9)
Correlation	0.9937	0.9532	0.9867
RMSE, cm^−2^	0.0947	0.1421	0.0942

According to the correlation and RMSE values, diffusion theory model showed the closest relationship followed by anisotropic Monte Carlo simulation. Diffusion theory model obtained slightly better correlation, while Monte Carlo simulation reached slightly lower RMSE value.

## Additional information

• PENELOPE2014, A Code System for Monte-Carlo Simulation of Electron and Photon Transport https://www.oecd-nea.org/tools/abstract/detail/nea-1525
• Scott Prahl: Monte Carlo Light Scattering Programs https://omlc.org/software/mc/
• MCBackscattering: Monte Carlo Simulation for Surface Backscattering https://cran.r-project.org/package=MCBackscattering

## Conflict of Interest

Author declare no conflict of interest

## Appendix Supplementary materials

Image, application 1
