# Supplementary material for: Laser induced diffuse reflectance imaging – Monte Carlo simulation of backscattering measured on the surface
Source: MethodsX. 2020 Jun 18;7:100958. doi: 10.1016/j.mex.2020.100958 (PMC7327276; doi:10.1016/j.mex.2020.100958)
Supplement: Supplementary file 1 [file mmc1.pdf]

MethodsX

Laser induced diffuse reflectance imaging - Monte Carlo simulation of backscattering measured on the surface

--Manuscript Draft--

**Manuscript Number:**	MEX-D-19-00331R1
**Article Type:**	Method article
**Section/Category:**	Agricultural and Biological Sciences
**Keywords:**	photon trajectory;<br>light interaction;<br>spatial multispectral imaging
**Corresponding Author:**	Laszlo Baranyai, PhD<br>Szent Istvan University<br>Budapest, HUNGARY
**First Author:**	Laszlo Baranyai, PhD
**Order of Authors:**	Laszlo Baranyai, PhD
**Abstract:**	The Monte Carlo simulation algorithm of photon trajectory computation is implemented in object oriented R code. Diffuse reflectance, also called backscattering, is modeled in semi-infinite homogeneous media. Spatial photon flux leaving the surface of the media is collected. The profile of intensity along radii relative to the incident point is used to simulate measurement of computer vision systems. Four optical parameters of the media are used: absorption coefficient, scattering coefficient, anisotropy factor and refractive index. Five parameters are used to describe configuration of the vision system: number of photons, radius of circular light beam, limiting energy level of photons, radius of observed area, spatial resolution of the vision system.<br><br>The incident angle of the light beam is included in the photon launch procedure. Initial direction is typically assumed to be normal with x,y,z coordinates of 0,0,1. In the proposed modification, initial move vector is calculated based on the incident angle and refractive index of the media. Additionally, elliptic distortion of the circular light beam on the surface is calculated based on the incident angle.<br><br>Photon flux leaving media through the surface is corrected with Lambertian method to measure intensity captured by an imaging device in normal position.<br><br>The software implementing the method is written in R language, the R code is available as standard package.
**Response to Reviewers:**	Answer to reviewer is also attached as single file.<br><br>I would like to thank the time and job of the reviewer. I appreciate very much the positive comments and remarks that help to improve the manuscript. There were also suggestions for future development of the software.<br><br>Changes in the text are marked with red color.<br><br>Answers to questions:<br><br>1., Thank you for the suggestion, corrected.<br><br>2., Thank you for the comment. Yes, it is technically possible. The sentence was removed and this point was modified.<br><br>I had experience with semi transparent mirror and the glare was disturbing in our measurements. In the literature of the field of agriculture and food science, experimental setups commonly use non 0°/0° adjustment. Incident angle is usually

Powered by Editorial Manager® and ProduXion Manager® from Aries Systems Corporation

reported to be in the range of 10-20°. This was the reason of the statement.

3., Thank you for the suggestion. Reference materials were provided with selected single wavelength and optical parameters are known only for that one. They were calibrated on 680 nm. Yes, I agree, it would be nice to have values for other wavelengths too, but in the laboratory we had no instrument to measure them.

4., Thank you for the comment. The code was tested on different computers and one example benchmarking is shown as “Single core process simulation of 106 photons took approximately 2 h on Intel i3 processor (3.83 GFLOPS/core).”
Yes, parallel running was considered to accelerate simulation. The parallel package of R was reported to have issue with random number generation, due to the copy of the whole environment for parallel processes. New version is told to solve the issue but it was not tested yet with this code. The next version of this R class hopefully will provide this option. Presently R code can run separate threads from consoles to decrease computation time.

5., Thank you for the suggestion. Both setup (Table 4) and randomize (Table 5) functions are presented in the text. They are made to use the advantage of R language and generate random numbers in advance to accelerate computation.

6., Thank you for pointing on this issue. Yes, text was modified accordingly.

7., The length of one segment is computed in move function as

```
# travel length
d <- -1.0*log(myObject$rmv[myObject$midx])
```

Therefore the length is likely no 1.0

8., Thank you for pointing out this mistake. The value was 1.35. It was estimated in the optical laboratory based on the critical angle (one laser module was used for measurement). The refractive index was not provided by the company.

9., Thank you very much for the suggestion!

10., Thank you for the comment. The simulation is computing trajectories in semi infinite media with plain surface presently. The surface curvature and acceptance angle of zoom lens can be considered in post processing. Examples for fruits were published in:
- Lu, R. (2009). Spectroscopic technique for measuring the texture of horticultural products: spatially resolved approach, In: Optical monitoring of fresh and processed agricultural crops, Zude, M. (Ed.) 391-423, CRC Press, ISBN 978-1-4200-5402-6, Boca Raton, USA.
- Lu, R. & Peng, Y. (2007). Development of a multispectral imaging prototype for real-time detection of apple fruit firmness. Optical Engineering, Vol. 46, No. 12, December 2007, 123201
- Peng, Y. & Lu, R. (2008). Analysis of spatially resolved hyperspectral scattering images for assessing apple fruit firmness and soluble solids content. Postharvest Biology and Technology, Vol. 48, No. 1, April 2008, 52-62, ISSN 0925-5214

Yes, I would like to continue development of the package with post processing functions too.

Powered by Editorial Manager® and ProduXion Manager® from Aries Systems Corporation

Cover Letter

To:
Editors
MethodsX

Dear Editors,

thank you for evaluating the manuscript “Laser induced diffuse reflectance imaging I. - Monte Carlo simulation of backscattering measured on the surface”. The reviewer comments and suggestions were considered, all changes in text are marked in red. The proposed R package is approved at CRAN system and available for anyone. Due to the coding style requirements, some feedback messages were changed in the R code.

This method and software package has not been published previously and not submitted parallel to any journal. Author declare no conflict of interest.

The manuscript was planned to be co-submission with image processing method, but those results need confirmation with another experiment. Presently I can upload manuscript as direct submission.

In case the manuscript is accepted, I wish to present color figures only in the online electronic version.

Sincerely,

László Baranyai

Answer to reviewers

I would like to thank the time and job of the reviewer. I appreciate very much the positive comments and remarks that help to improve the manuscript. There were also suggestions for future development of the software.

Changes in the text are marked with red color.

**Comment/remark/question**	**Answer**
1. in the abstract this sentence is somehow confusing: "The profile of intensity along radii relative to the incident point is to simulate measurement of computer vision systems." - perhaps "is used" or "may be used"?	Thank you for the suggestion, corrected.
2. Still in the abstract, "Since imaging device and light source cannot be placed in the same direction, incidence differs from normal". This is repeated again in the text. Actually, it is possible to have both incidence and observation in the normal, by using a beam splitter. The unavoidable glare at the beam splitter surface may be removed through a background subtraction. For the background photo one may substitute the turbid media by a perfectly black absorber while keeping the beam splitter in place. So, my point is that the author should not be so affirmative in that the geometry 0º/0º geometry is not possible.	Thank you for the comment. Yes, it is technically possible. The sentence was removed and this point was modified.<br><br>I had experience with semi transparent mirror and the glare was disturbing in our measurements. In the literature of the field of agriculture and food science, experimental setups commonly use non 0°/0° adjustment. Incident angle is usually reported to be in the range of 10-20°. This was the reason of the statement.
3. Figure 2: it would be interesting to have the optical parameters at the other wavelengths, namely 532, 660, 785 and 830 nm	Thank you for the suggestion. Reference materials were provided with selected single wavelength and optical parameters are known only for that one. They were calibrated on 680 nm. Yes, I agree, it would be nice to have values for other wavelengths too, but in the laboratory we had no instrument to measure them.
4. It would be important to give an idea of the execution time. In my laptop (Intel Core i7-8750H CPU @ 2.20GHz × 12) it took me about 70 minutes to run the simulation with one million photons. So, roughly, 1 million photons per hour. Did the author considered the possibility of parallelize the code? I am not sure it could be used here, but presumably yes. Each photon is independent, so the calculation of the paths could be distributed by the available cores. And there are packages to perform parallelization in R. So, I would like to know the author's opinion on that matter.	Thank you for the comment. The code was tested on different computers and one example benchmarking is shown as “Single core process simulation of 10^6 photons took approximately 2 h on Intel i3 processor (3.83 GFLOPS/core).”<br>Yes, parallel running was considered to accelerate simulation. The parallel package of R was reported to have issue with random number generation, due to the copy of the whole environment for parallel processes. New version is told to solve the issue but it was not tested yet with this code. The next version of this R class hopefully will provide this option. Presently R code can run separate threads from consoles to decrease computation time.
5. I understand that the author explained in detail only those functions with changes relative to the MCML code. However, I suggest that a few lines about the Setup function could help the reader to understand the whole picture.	Thank you for the suggestion. Both setup (Table 4) and randomize (Table 5) functions are presented in the text. They are made to use the advantage of R language and generate random numbers in advance to accelerate computation.
6. The following sentences probably still have the numbers of a previous manuscript version:<br>"The implemented code can be used according to Table 1-3" → you mean Table 2?<br>"Photon flux can be retrieved as intensity profile by Export function (Table 2-3)" →you mean Table 3?	Thank you for pointing on this issue. Yes, text was modified accordingly.
7. In the code, in the following line	The length of one segment is computed in move function

# Lambertian correction to normal direction<br>lcc <- abs(myObject$w) / sqrt(myObject$u^2 + myObject$v^2 + myObject$w^2)<br><br>Is that really necessary, since myObject$u^2 + myObject$v^2 + myObject$w^2 = 1?	as<br>`# travel length`<br>`d <- -1.0*log(myObject$rmv[myObject$midx])`<br><br>Therefore the length is likely no 1.0
8. In the simulation (Table 7) the refractive index is really 1.3, less than water? What material was that? And how did PDW Analytics characterized its optical parameters? (this is relevant in this context)	Thank you for pointing out this mistake. The value was 1.35. It was estimated in the optical laboratory based on the critical angle (one laser module was used for measurement). The refractive index was not provided by the company.
9. Table 8: If DA is slightly better, then this is because the removed pixels (those over saturated) were precisely those where DA could fail. My suggestion for a future work: rewrite the code for transmission and use a thin slab phantom. In this way all the pixels will be used and the advantage of MC close to r=0 will be obvious.	Thank you very much for the suggestion!
10. Shouldn't the angular field of view of the camera be taken into account? Only photons escaping the media within some angular cone (which depends on the location of the escape point) will be collected by the camera. Maybe I did not understood it correctly, but I could not spot the code lines to do that angular selection of the photons escaping the surface.	Thank you for the comment. The simulation is computing trajectories in semi infinite media with plain surface presently. The surface curvature and acceptance angle of zoom lens can be considered in post processing. Examples for fruits were published in:<br>- Lu, R. (2009). Spectroscopic technique for measuring the texture of horticultural products: spatially resolved approach, In: *Optical monitoring of fresh and processed agricultural crops*, Zude, M. (Ed.) 391-423, CRC Press, ISBN 978-1-4200-5402-6, Boca Raton, USA.<br>- Lu, R. & Peng, Y. (2007). Development of a multispectral imaging prototype for real-time detection of apple fruit firmness. *Optical Engineering*, Vol. 46, No. 12, December 2007, 123201<br>- Peng, Y. & Lu, R. (2008). Analysis of spatially resolved hyperspectral scattering images for assessing apple fruit firmness and soluble solids content. *Postharvest Biology and Technology*, Vol. 48, No. 1, April 2008, 52-62, ISSN 0925-5214<br><br>Yes, I would like to continue development of the package with post processing functions too.

Method article template

Click here to access/download;Method article template;MonteCarlo_Backscattering_MethodsX_revision.pdf

Meta-Data (Required for the transfer of your article to MethodsX – will not be typeset)

***Title:** *Max. 20 words.*<br>• *A good title should contain the fewest possible words that adequately describe the content of a paper.*	*Laser induced diffuse reflectance imaging - Monte Carlo simulation of backscattering measured on the surface*
***Authors:**	*László Baranyai*[1]
***Affiliations:**	[1]*Leibniz Institute for Agricultural Engineering and Bioeconomy (ATB), Germany*
***Contact email:** *Include institutional email address of the corresponding author*	*lbaranyai@atb-potsdam.de*
Co-authors**: *full names and e-mails.*<br>[NOTE: it is the corresponding authors responsibility to inform all co-authors if submitting as a companion paper to a research article]***	
***Keywords:** *At least 3 keywords.*<br>• *There is no limit on the no. of keywords you can list.*<br>• *Please remember that effective keywords should not repeat words appearing in your title, and should be neither too general nor too narrow.*	*photon trajectory,*<br>*light interaction,*<br>*spatial multispectral imaging*
***SECTION:**<br>• *Agricultural and Biological Sciences*<br>• *Biochemistry, Genetics and Molecular Biology*<br>• *Chemical Engineering*<br>• *Chemistry*<br>• *Computer Science*<br>• *Earth and Planetary Sciences*<br>• *Economics and Finance*<br>• *Energy*<br>• *Engineering*<br>• *Environmental Science*<br>• *Immunology and Microbiology*<br>• *Materials Science*<br>• *Medicine and Dentistry*<br>• *Neuroscience*<br>• *Pharmacology, Toxicology and Pharmaceutical Science*<br>• *Physics and Astronomy*<br>• *Psychology*<br>• *Veterinary Science and Veterinary Medicine*	Agricultural and Biological Sciences<br>Computer Science
**Submission:**	Direct submission

Method Article

*Title: Laser induced diffuse reflectance imaging - Monte Carlo simulation of backscattering measured on the surface
*Authors: László Baranyai[1]
*Affiliations: [1]Leibniz Institute for Agricultural Engineering and Bioeconomy (ATB), Germany
*Contact email: lbaranyai@atb-potsdam.de
*Keywords: photon trajectory, light interaction, spatial multispectral imaging

ABSTRACT

***Abstract:** The Monte Carlo simulation algorithm of photon trajectory computation is implemented in object oriented R code. Diffuse reflectance, also called backscattering, is modeled in semi-infinite homogeneous media. Spatial photon flux leaving the surface of the media is collected. The profile of intensity along radii relative to the incident point is used to simulate measurement of computer vision systems. Four optical parameters of the media are used: absorption coefficient, scattering coefficient, anisotropy factor and refractive index. Five parameters are used to describe configuration of the vision system: number of photons, radius of circular light beam, limiting energy level of photons, radius of observed area, spatial resolution of the vision system.

- *The incident angle of the light beam is included in the photon launch procedure. Initial direction is typically assumed to be normal with x,y,z coordinates of 0,0,1. In the proposed modification, initial move vector is calculated based on the incident angle and refractive index of the media. Additionally, elliptic distortion of the circular light beam on the surface is calculated based on the incident angle.*
- *Photon flux leaving media through the surface is corrected with Lambertian method to measure intensity captured by an imaging device in normal position.*
- *The software implementing the method is written in R language, the R code is available as standard package.*

SPECIFICATIONS TABLE

**Subject Area**	*Agricultural and Biological Sciences*<br>*Computer Science*
**More specific subject area:**	*Optical properties of biological tissue*
**Method name:**	*Monte Carlo simulation of diffuse reflectance measured on the surface*
**Name and reference of original method**	*The code presented in this paper implements stochastic Monte Carlo algorithm to simulate light distribution in media and its measurement on the surface. The computational methods are inspired by the literature and primarily:*<br>- Francesc Salvat: PENELOPE-2014 - A Code System for Monte Carlo Simulation of Electron and Photon Transport - Workshop Barcelona, Spain 29 June-3 July 2015 (NEA/NSC/DOC(2015)3)<br>- Jacques, S.L., 1998. Light distributions from point, line and plane sources for photo-chemical reactions and fluorescence in turbid biological tissues. Photochemistry and Photobiology 67 (1), 23–32.
**Resource availability**	*Object oriented R code (www.r-project.org) was written and an R package has been created.*

*Method details

The name Monte Carlo (MC) indicates stochastic behavior of computation. The simulation of diffuse reflectance, also known as backscattering, follows photon pathways inside medium and summarizes photon flux leaving the surface. The simulation assumes semi-infinite homogeneous media and an imaging device above the surface to collect photons and measure spatial intensity distribution. Detailed description of the photon trajectory computation algorithm and software libraries are available in Fortran language (with the name of PENELOPE) [1,2] and ANSI C language (with the name of MCML and CONV) [3,4]. This implementation uses variable names similar to the ANSI C code.

Computer vision systems measure spatial intensity distribution relative to the incident point of the light beam [5,6]. The light beam injects photons into the media and surrounding area got illuminated by diffuse reflectance. Intensity is typically measured in concentric rings of 1 pixel width (Fig. 1). The captured intensity profile is very similar to the result of Monte Carlo simulation. Monte Carlo simulation can be used in inverse modeling. Estimation functions, established based on the observed results of simulation, can make predictions in vision systems [7,8].

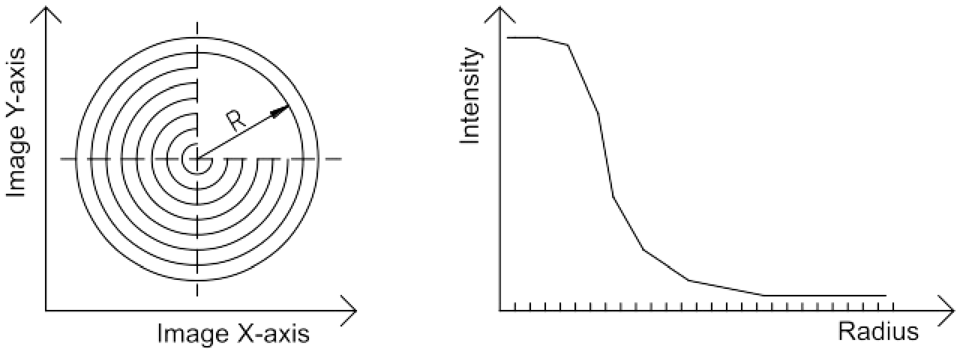

Fig. 1. Radial averaging pattern of concentric rings (left) and typical shape of acquired intensity profile (right).
R is the radius measured from incident point.

The measured photon flux and the shape of the intensity profile (Fig. 1) depends on the optical properties of the media. These parameters are μ_a absorption coefficient (cm^{-1}), μ_s scattering coefficient (cm^{-1}), *g* anisotropy factor and *n* refractive index of media. The reduced scattering coefficient (Eq. 1) is reported in many publications as simplified parameter of the theoretical model [9].

$$\mu_s' = (1-g)\mu_s \qquad \text{(Eq. 1)}$$

This simplification makes validation more difficult, since many different combinations of *g* and μ_s can result the same μ_s' reduced scattering coefficient. Media could be assumed isotropic ($g = 0$) but biological materials are reported to scatter forward $g > 0.6$ [10]. Additionally, absorption and scattering coefficients may differ by wavelength. The effect of wavelength on optical measurement is demonstrated in Fig. 2, where the same object can be observed with diffuse reflectance induced at multiple wavelengths.

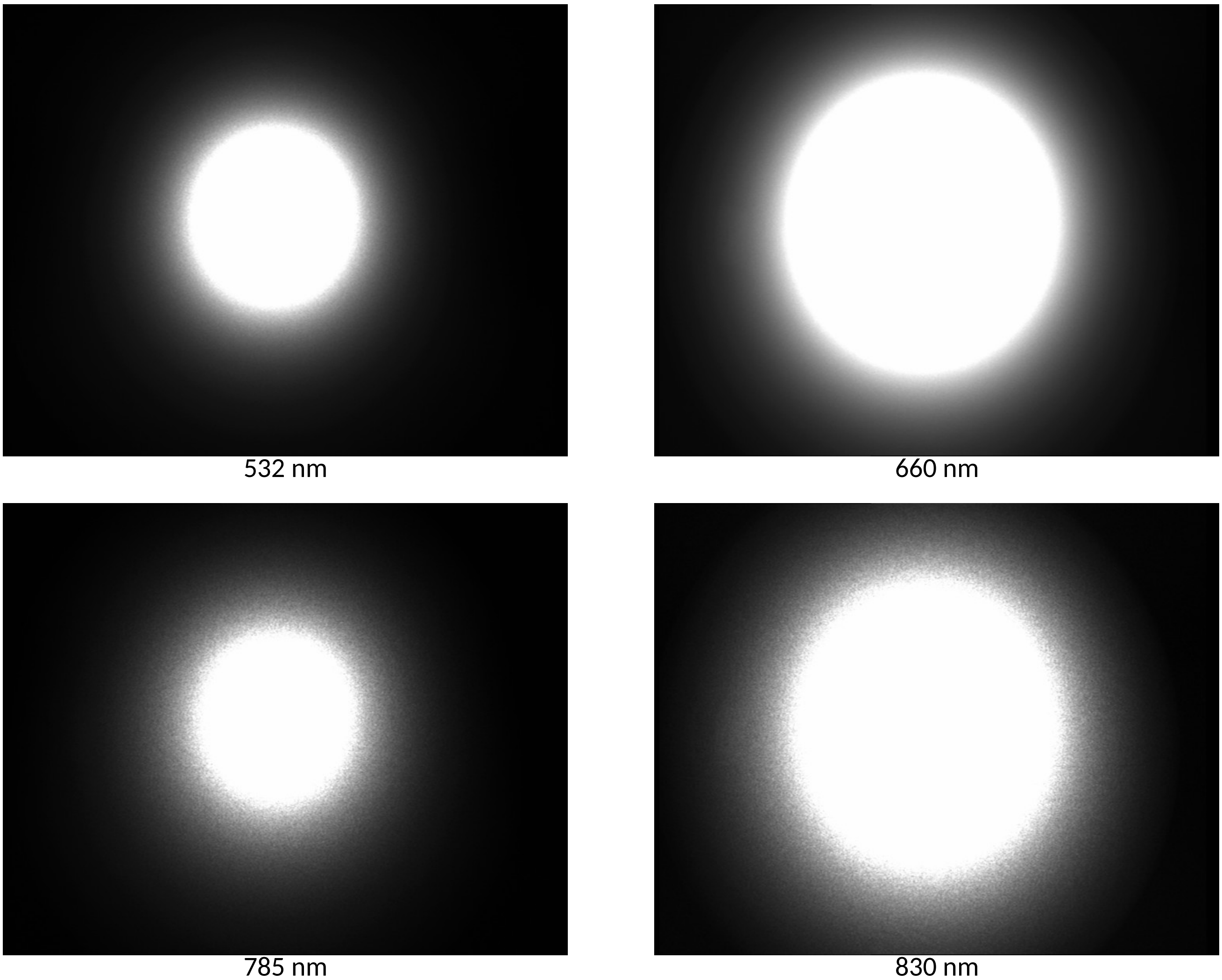

Fig. 2. Laser induced diffuse reflectance of the same object at different wavelengths.
Reference material of μ_a = 0.1056 cm^{-1} and μ_s' = 2.529 cm^{-1} at 680 nm

The computational parameters of the simulation include number of photons, radius of circular light beam, limiting energy level of photons, radius of observed area, spatial resolution of the vision system. The number of photons significantly affects computation time, therefore the minimum sufficient number shall be adjusted. The noise of the calculated intensity profile decreases with increasing number of photons. Based on the power of the light source and wavelength of emitted light, required number of photons can be calculated using Planck's law (Eq. 2):

$$E = h v = h \frac{C_0}{n \lambda} \qquad \text{(Eq. 2)}$$

where E is the photon energy, h is the Planck's constant, v is the frequency, C_0 is the velocity of light in vacuum, λ is the wavelength of light and n is the refractive index of media. For example, 1 s light pulse of 670 nm wavelength of 3 mW power in media of n = 1.4 result in 1.42×10^{16} photons. This number can decrease if integration time of the imaging device of computer vision system was considered. Based on time resolved calculations, the 1 ns pulse length was found to be sufficient for simulation of light penetration into apple [5].

The hardware parameters of the computer vision system are constant for the same setup, such as image resolution, beam radius. The parameter limiting energy shall be low enough to allow drop low energy photons without significant computation error. The simulation can be initialized with optical parameters of media and parameters of the computer vision system. The R code to load package and initialize simulation object is presented in Table 1. Absorption and scattering coefficients are expected in cm^{-1} unit.

Table 1. Loading library and configuration of simulation object MCBS in R language.

```
## Load library
library("MCBackscattering")

## Apple tissue properties according to
## Qin and Lu (2006) DOI: 10.13031/2013.20862
cfgMedia <- c(
0.63,  # absorption 1/cm, 670 nm
30,    # scattering 1/cm, 670 nm
0,     # isotropic tissue assumed
1.4)   # refractive index

## Computer vision system and simulation parameters
cfgSimulation <- c(
1e7,   # 10 million photons
0.05,  # 1 mm diameter (0.05 cm radius) laser light beam
1e-9,  # limiting energy level
3,     # 3 cm radius is computed
0.01)  # 0.01 cm/pixel resolution

apple <- MCBS(cfgMedia,cfgSimulation)
```

Running simulation with this implementation does not require additional preparation. One function is made to perform all computations (Table 2) and result can be presented on chart or extracted as table.

Table 2. Usage of simulation function in R language. Code also shows profile on chart and saves data into file.

```
## Run simulation with default incident angle
apple <- Simulation(apple)

## Show intensity profile
Chart(apple)

## Save results into file with data table
write.table(Export(apple),"apple.dat")
```

Low number of photons in simulation obtain significant noise on the intensity profile. Low intensity values far from incident point may have higher noise due to the lower number of photons in that area. According to our experiences, the minimum recommended number of photons is 10^6. The effect of the number of photons is presented on Fig. 3. Simulation of absorbed energy inside media can require less photons compared to diffuse reflection on surface, because the number of trajectories leaving the media on observed area is lower.

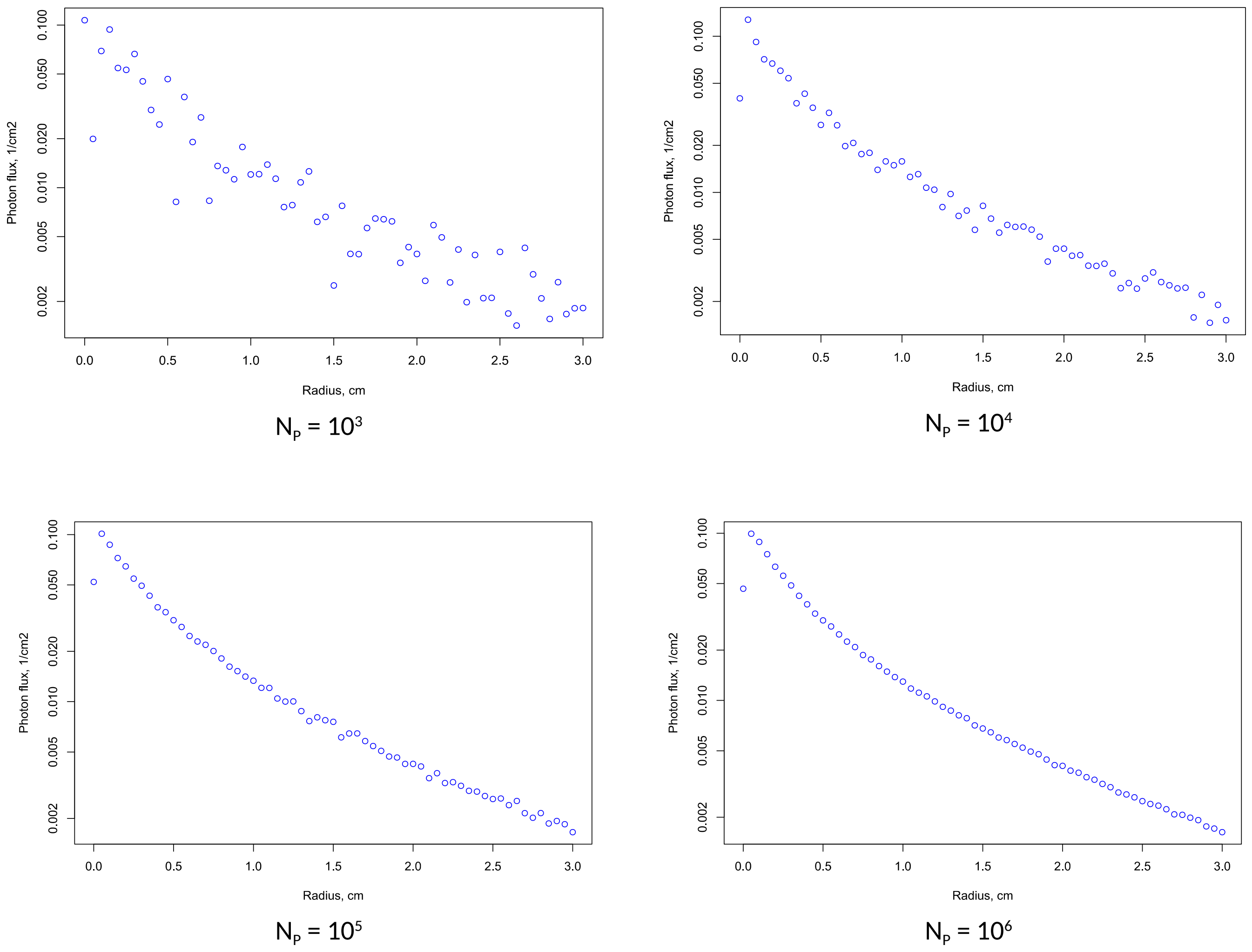

Fig. 3. Effect of the number of photons (N_P) on simulation result (μ_a = 0.1056 cm^{-1}, μ_s = 2.529 cm^{-1}, g = 0, n = 1.35)

The simulation function (Table 2) has an optional second parameter, the incident angle of light beam. This value is used when photon is launched into media. If parameter is missing, default value of 0° is assumed. The normal position, above incident point, is occupied by the camera or other imaging device. Therefore, light source cannot be placed in the same direction. Measurement geometry of low incident angle is recommended to utilize large amount of photons. On the other hand, incident angle should be large enough to avoid direct reflection into the camera. The incident angle of 10–20° is recommended. The incident angle can be used with simulation function (Table 3).

Table 3. Usage of simulation function in R language with 15° incident angle. Code also saves data into table.

```
## Run simulation with 15 deg incident angle
apple <- Simulation(apple,15)

# Save results into file with data table
write.table(Export(apple),"apple.dat")
```

Computational methods presented in literature use normal direction, perpendicular to surface [1-4,9]. Due to the limitations of hardware setup, this should be reconsidered.

Setup function (Table 4) was made to initialize computation at the beginning of the simulation. Container is created for results and derived parameters, such as albedo and critical angle, are calculated. The computation of simulation function may take long since it follows all photon trajectories. In order to accelerate procedure, maximum trajectory length and random launch position of all photons are adjusted, as well.

Table 4. Setup function implementation in R language to prepare containers, calculate parameters.

```
## Initialize computation
Setup <- function(myObject) { UseMethod("Setup",myObject) }
Setup.MCBS <- function(myObject)
```

```
{
  # container for intesity profile
  myObject$heat <- rep(0,1 + round(myObject$radius / myObject$rpx))
  # transport albedo = intensity decrease after interaction event
  myObject$albedo <- myObject$mu_s / (myObject$mu_s + myObject$mu_a)
  # specular reflection
  myObject$rs <- ( (myObject$n - 1) / (myObject$n + 1) )^2
  # critical angle
  myObject$cangle <- sqrt(1.0 - 1.0/(myObject$n^2))
  # maximum trajectory length in events
  myObject$MAXLEN <- round( log(myObject$limit)/log(myObject$albedo) )+1
  # beam start position, radius and angle
  myObject$rbr <- myObject$beamr*sqrt(runif(myObject$photons))
  myObject$rba <- runif(myObject$photons,min=0,max=2*pi)
  # reset summary values
  myObject$rd <- 0
  myObject$bit <- 0

  return(myObject)
}
```

A randomize function (Table 5) was made with the purpose to accelerate computation and generate random numbers in advance for each photon. Computation later can refer to the prepared vectors of random numbers instead of calling the random number generation function each time.

Table 5. Randomize function implementation in R language to generate random numbers for single photon.

```
## Fill random data for single photon trajectory
Randomize <- function(myObject) { UseMethod("Randomize",myObject) }
Randomize.MCBS <- function(myObject)
{
  # maximum trajectory length is computed to MAXLEN
  # random numbers are selected from uniform distribution
  # move length, 0-1
  myObject$rmv <- runif(myObject$MAXLEN)
  # absorption roulette, 0-1
  myObject$rabs <- runif(myObject$MAXLEN)
  # new direction after scattering, from -1 to +1
  myObject$rx1 <- runif(myObject$MAXLEN,min=-1,max=1)
  myObject$rx2 <- runif(myObject$MAXLEN,min=-1,max=1)
  # Heyney-Greenstein phase function random variable, 0-1
  myObject$rmu <- runif(myObject$MAXLEN)

  return(myObject)
}
```

Photon trajectory computation has five steps:

- launch photon
- move in media
- bounce if photon leaves media through surface
- absorb energy
- scatter in media

Common computational method is modified in launch and bounce. Launch is considering incident angle and its effects, bounce collects photon weights on surface. All steps are available as separate functions to allow users build customized procedure.
The launch function has an optional second parameter, the incident angle. Initial direction inside media is calculated on the basis of the incident angle and refractive index (Eq. 3).

$$\phi_2 = \sin^{-1}\left(\frac{\sin\phi_1}{n_2}\right) \qquad \text{(Eq. 3)}$$

where ϕ_1 is the incident angle in air, ϕ_2 is the refracted angle in media and n_2 is the refractive index of the media. When photon is emitted from light source, its weight is 1. The photon weight decreases first time during interaction with surface. Photons entering media are usually considered to have normal start direction (u,v,w) = (0,0,1). This direction is changed according to the incident

angle. In media of n = 1.4, start angle is in the range of 7.12° – 14.14° for recommended incident angle range of 10° – 20°. Additionally, due to the rotation of the incident light beam, its circular cross section is elliptic on the surface. This elliptical distortion is calculated on the launch position. The start position of the photon on the surface (x,y,z=0) is calculated randomly within the area of the circular light beam, using uniform distribution [11]. Elliptic distortion affects this position with the y coordinate. The R language implementation of launch function is presented in Table 6.

Table 6. Launch function implementation in R language to start photon trajectory from boundary.

```
## Launch single photon within beam
# Parameter:
# iAngle = incident angle, default = 0 deg (relative to normal)
Launch <- function(myObject,iAngle=0) { UseMethod("Launch",myObject) }
Launch.MCBS <- function(myObject,iAngle=0)
{
  # initial photon weight
  myObject$weight <- 1.0 - myObject$rs
  # internal angle after refraction
  myAngle <- asin(sin(iAngle*pi/180)/myObject$n)
  # incident direction
  myObject$u <- 0
  myObject$v <- sin(myAngle)
  myObject$w <- cos(myAngle)
  # start position
  myObject$x <- myObject$rbr[myObject$idx] * cos(myObject$rba[myObject$idx])
  myObject$y <- myObject$rbr[myObject$idx] * sin(myObject$rba[myObject$idx]) / cos(myAngle)
  myObject$z <- 0

  return(myObject)
}
```

During simulation, photons are identified with index number `idx` and trajectory moves for each photon are identified with index number `midx`. These indices are used to access containers of random numbers, such as launch position polar coordinates `rbr` for radius and `rba` for angle (Table 4,6). Bulk generation of random numbers is done for optimization of computation.

The second modified function compared to common algorithm is the bounce method. Photons leaving the surface are collected in this step. Photon position (x,y,z) is out of media if z < 0. When this occurs, moving direction (u,v,w) also points out of the media with w < 0. Moving direction is compared to critical angle to decide whether internal reflection happens or photon can leave media. Fresnel reflection is calculated to correct photon weight. Additionally, Lambertian correction is performed modeling the camera or imaging device in normal position above the surface. Photon flux is collected in vector `heat`, with spatial resolution. According to the R language, the first element of the vector has index 1, which belongs to the incident point of radius r = 0. Each element of the vector represent photon flux leaving the surface in the area of concentric ring around the incident point. The source code of the bounce function implemented in R language is presented in Table 7.

In order to receive the normalized photon flux in cm^{-2} unit, vector elements are divided by the corresponding surface area during post processing. The area of the concentric rings can be calculated using two equivalent equations (Eq. 4-5).

$$A=\pi\left((r+dr)^2-r^2\right)=\pi\left(2r\,dr+dr^2\right) \quad \text{(Eq. 4)}$$

$$A=2\pi\left(r+\frac{dr}{2}\right)dr \quad \text{(Eq. 5)}$$

Where *A* is the area of the ring of *dr* width and *r* is the inner radius of the ring. The form of Eq. 5 is commonly used, but equations are equivalent and also calculated with similar speed. The R implementation is using formula Eq. 4.

Table 7. Bounce function implementation in R language to collect surface photon flux.

```
## Bounce interaction with surface
Bounce <- function(myObject) { UseMethod("Bounce",myObject) }
Bounce.MCBS <- function(myObject)
{
  myObject$w <- -1*myObject$w
  myObject$z <- -1*myObject$z
  # check for internal reflection, then nothing to do
  if (myObject$w > myObject$cangle) {
    t <- sqrt(1.0-(1.0-myObject$w^2)*myObject$n^2)
```

```
    temp1 <- (myObject$w - myObject$n*t)/(myObject$w + myObject$n*t)
    temp <- (t - myObject$n*myObject$w)/(t + myObject$n*myObject$w)
    # Fresnel reflection
    rf <- (temp1*temp1+temp*temp)/2.0
    myObject$rd <- myObject$rd + (1.0-rf) * myObject$weight
    # collect leaving photons by radius
    # Lambertian correction to normal direction
    lcc <- abs(myObject$w) / sqrt(myObject$u^2 + myObject$v^2 + myObject$w^2)
    lcc <- myObject$n * sqrt(1.0-lcc^2)
    if (lcc^2 > 1) {
      # failsafe check
      lcc <- 0
    } else {
      lcc <- sqrt(1.0 - lcc^2)
    }
    # compute radius
    r <- sqrt(myObject$x^2 + myObject$y^2)
    r <- round(r / myObject$rpx) + 1
    if (r <= length(myObject$heat)) {
      myObject$heat[r] <- myObject$heat[r] + lcc * (1.0-rf) * myObject$weight
    }
    # continue travel inside
    myObject$weight <- myObject$weight - (1.0-rf) * myObject$weight;
  }

  return(myObject)
}
```

Computations are accelerated to decrease simulation runtime. Trigonometric functions are substituted where it is possible using the following equation (Eq. 6).

$$\cos^2\phi+\sin^2\phi=1 \text{ and } \sin\phi=\sqrt{1-\cos^2\phi}\,;\ \cos\phi=\sqrt{1-\sin^2\phi} \qquad \text{(Eq. 6)}$$

The simulation uses a lot of random numbers to launch photons and calculate their trajectory. Random numbers are generated in bulk for optimization. Start polar coordinates are generated in a vector with the length of the number of photons (Table 4). Trajectory random variables are generated in vectors with the length calculated from limiting energy and transport albedo (Table 5). Table 8 presents the random vectors used int the R implementation.

Table 8. Random vectors of R implementation of Monte Carlo simulation.

Vector name	Value range	Created by function	Used by function	Comment
`rbr`	0 – r_b*	Setup	Launch	Launch position polar coordinate, radius in light beam
`rba`	0 – 2π	Setup	Launch	Launch position polar coordinate, angle
`rmv`	0 – 1	Randomize	Move	Length of straight segment of trajectory
`rabs`	0 – 1	Randomize	Absorb	Photon survival decision is made after interaction
`rx1, rx2`	-1 – +1	Randomize	Scatter	New direction coordinates after scattering
`rmu`	0 – 1	Randomize	Scatter	Anisotropic scattering by Henyey-Greenstein function

* r_b is the radius of light beam

The implemented code can be used according to presented sample (Table 2). Only two functions are required to perform simulation, the `MCBS` object constructor and `Simulation` to perform computation. Photon flux can be retrieved as intensity profile by `Export` function (Table 3). If one would like to make customized procedure, functions are available and `Simulation` function can be used as template.

Due to the interpreted R codes, computation consumes more time than compiled C software. Parallel processing may utilize multiple cores of processors on POSIX computer systems. Single core process simulation of 10^6 photons took approximately 2 h on Intel i3 processor (3.83 GFLOPS/core).

Method validation

Reference material of known optical properties was produced by PDW Analytics GmbH (Potsdam, Germany). The absorption coefficient and reduced scattering coefficient of solid phantom are known as μ_a = 0.1056 cm^{-1} and μ_s' = 2.529 cm^{-1} at 680 nm. The

Monte Carlo simulation was repeated twice and used different combinations of anisotropy factor and scattering coefficient (resulting the same reduced scattering coefficient). Simulation parameters are listed in Table 9. The diffusion theory model [9] was also computed as reference. As a result of changing optical properties, rotation of simulated profiles can be observed [5,6]. Comparing the two simulations, results of isotropic media (g = 0) fit better to diffusion theory model in terms of correlation and RMSE (root mean squared error).

Table 9. Monte Carlo simulation parameters and results of validation.

Parameter	Isotropic media (g = 0)	Anisotropic media (g = 0.9)
Absorption coefficient (μ_a, cm^{-1})	0.1056	0.1056
Scattering coefficient (μ_s, cm^{-1})	2.529	25.29
Anisotropy factor (g)	0	0.9
Refractive index	1.35	1.35
Number of photons	10^6	10^6
Beam radius, cm	0.05	0.05
Limiting energy level for photons	10^{-9}	10^{-9}
Observed radius, cm	3	3
Spatial resolution, cm/pixel	0.05	0.05
Correlation with diffusion model	0.9903	0.9529
RMSE with diffusion model, cm^{-2}	0.0534	0.1744

Backscattering images were recorded for the reference material at 660 nm wavelength (Fig. 2), using a laser module of 3 mW and a CCD camera (CV-A50IR, JAI Ltd., Japan) with zoom lens of 18-108 mm and f/2.5 (12VG1040 ASIR-SQ, Tamron Co. Ltd, Japan). The resolution of the images were 0.01205 cm/pixel. Images were recorded with 8 bit/pixel color depth, therefore pixel intensities ranged 0-255. All intensity profiles, measured and computed, were normalized to the range of 0-1 for comparison. The over saturated part near the incident point of the intensity profile was omitted from analysis. Comparison result of diffusion theory model as well as Monte Carlo simulation with measured signal is presented in Table 10.

Table 10. Comparison of computer vision system measurement with simulation results.

Parameter	Diffusion theory model	Monte Carlo simulation	
		Isotropic media (g = 0)	Anisotropic media (g = 0.9)
Correlation	0.9937	0.9532	0.9867
RMSE, cm^{-2}	0.0947	0.1421	0.0942

According to the correlation and RMSE values, diffusion theory model showed the closest relationship followed by anisotropic Monte Carlo simulation. Diffusion theory model obtained slightly better correlation, while Monte Carlo simulation reached slightly lower RMSE value.

Acknowledgements
The publication of this article was funded by the Open Access Fund of the Leibniz Association.

Conflict of interest
Author declare no conflict of interest

Additional information:

- PENELOPE2014, A Code System for Monte-Carlo Simulation of Electron and Photon Transport
  https://www.oecd-nea.org/tools/abstract/detail/nea-1525
- Scott Prahl: Monte Carlo Light Scattering Programs
  https://omlc.org/software/mc/
- MCBackscattering: Monte Carlo Simulation for Surface Backscattering
  https://cran.r-project.org/package=MCBackscattering

***References:**

1. Salvat, F., Fernández-Vaera, J.M., Acosta, E., Sempau, J.
   PENELOPE. A code system for Monte Carlo simulation of electron and photon transport.
   OECD Nuclear Energy Agency (2001), France, 234.
2. Salvat, F.
   PENELOPE-2014 - A Code System for Monte Carlo Simulation of Electron and Photon Transport
   Workshop Barcelona, Spain, 29 June-3 July 2015 (NEA/NSC/DOC(2015)3)

3. Wang, L., Jacques, S.L., Zheng, L.
MCML – Monte Carlo modeling of light trans- port in multi-layered tissues.
Computer Methods and Programs in Biomedicine, 47 (1995), 131–146
4. Wang, L., Jacques, S.L., Zheng, L.
CONV-convolution for responses to a finite diameter photon beam incident on multi-layered tissues.
Computer Methods and Programs in Biomedicine, 54 (1997), 141–150
5. Baranyai, L., Zude, M.
Analysis of Laser Light Migration in Apple Tissue by Monte Carlo Simulation
Progress in Agricultural Engineering Sciences, 4 (2008), 45–59
6. Baranyai, L., Zude, M.
Analysis of laser light propagation in kiwifruit using backscattering imaging and Monte Carlo simulation.
Computers and Electronics in Agriculture, 69 (2009), 33–39
7. Qin, J., Lu., R.
Monte Carlo simulation for quantification of light transport features in apples
Computers and Electronics in Agriculture, 68 (2009), 44--51
8. Mendoza, F., Lu, R., Cen, H.
Grading of apples based on firmness and soluble solids content using Vis/SWNIR spectroscopy and spectral scattering techniques
Journal of Food Engineering, 125 (2014), 59–68
9. Farrell, T.J., Patterson, M.S., Wilson, B.
A diffusion theory model of spatially resolved, steady-state diffuse reflectance for the noninvasive determination of tissue optical properties in vivo.
Medical Physics, 19(4) (1992), 879–888
10. Askoura, M.L., Vaudelle, F., L'Huillier, J.P.
Multispectral measurement of scattering-angular light distribution in apple skin and flesh samples
Applied Optics, 55(32) (2016), 9217–9225
11. Jacques, S.L.
Light Distributions from Point, Line and Plane Sources for Photochemical Reactions and Fluorescence in Turbid Biological Tissues
Photochemistry and Photobiology, 67(1) (1998), 23–-32

*Graphical Abstract

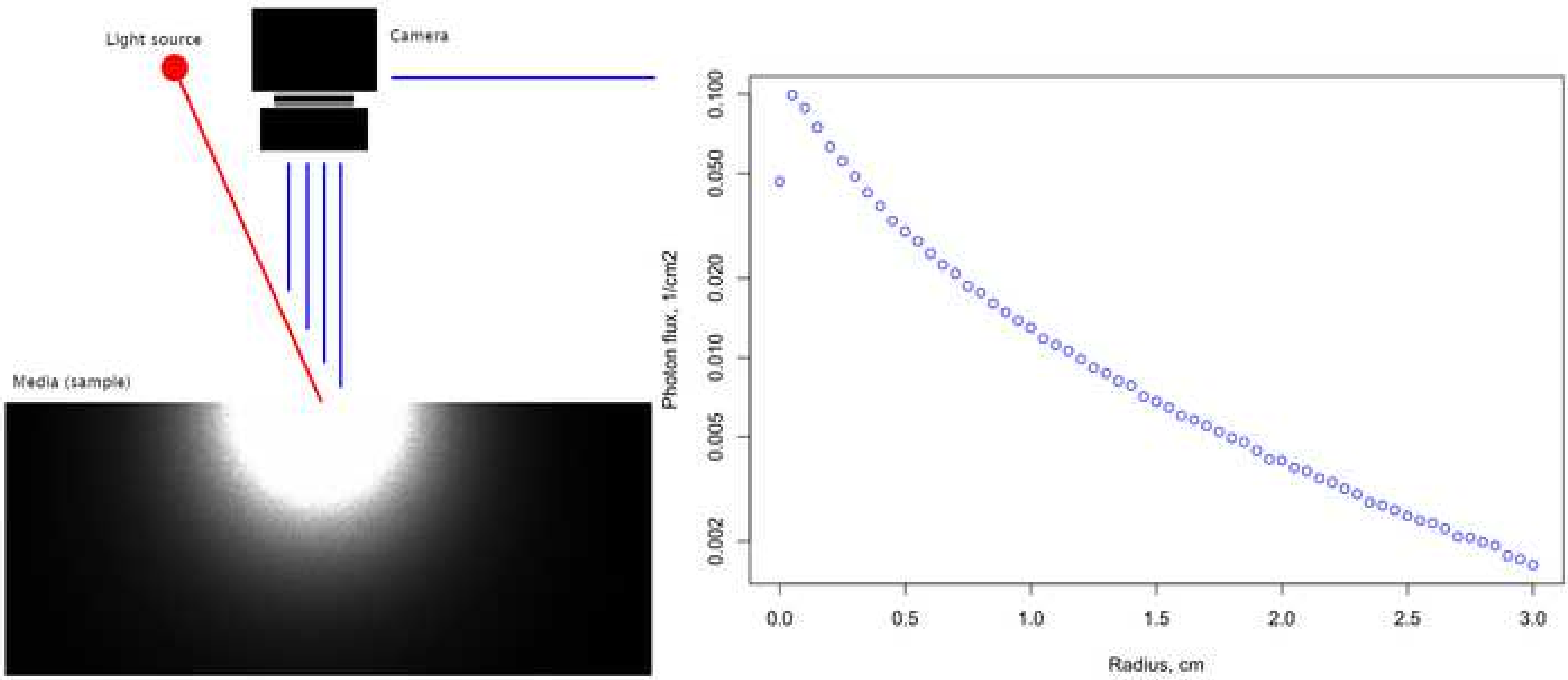

Click here to access/download
Supplementary Materials
MonteCarlo_R_object.R
